# Synthesis and Evaluation of LaBaCo_2−*x*_Mo*_x_*O_5+*δ*_ Cathode for Intermediate-Temperature Solid Oxide Fuel Cells

**DOI:** 10.3390/ma15175858

**Published:** 2022-08-25

**Authors:** Shoucheng He, Lanqing Zhang, Jiantao Cai, Xingyu Wu, Hanxi Sun, Tao Du

**Affiliations:** School of Materials Science and Engineering, Yancheng Institute of Technology, Yancheng 224051, China

**Keywords:** solid oxide fuel cell, perovskite cathode, thermal expansion coefficient, electrical conductivity, electrochemical performance

## Abstract

LaBaCo_2−*x*_Mo*_x_*O_5+*δ*_ (LBCM*x*, *x* = 0–0.08) cathodes synthesized by a sol-gel method were evaluated for intermediate-temperature solid oxide fuel cells. The limit of the solid solubility of Mo in LBCM*x* was lower than 0.08. As the content of Mo increased gradually from 0 to 0.06, the thermal expansion coefficient decreased from 20.87 × 10^−6^ K^−1^ to 18.47 × 10^−6^ K^−1^. The introduction of Mo could increase the conductivity of LBCM*x*, which varied from 464 S cm^−1^ to 621 S cm^−1^ at 800 °C. The polarization resistance of the optimal cathode LBCM0.04 in air at 800 °C was 0.036 Ω cm^2^, reduced by a factor of 1.67 when compared with the undoped Mo cathode. The corresponding maximum power density of a single cell based on a YSZ electrolyte improved from 165 mW cm^−2^ to 248 mW cm^−2^ at 800 °C.

## 1. Introduction

Energy is the driving force of human civilization and an important indicator of national economic development and people’s living standards. For a long time into the future, the world’s energy pattern will continue to be dominated by fossil energy sources such as coal, oil, and natural gas. Due to the non-renewable nature of fossil energy and the environmental pollution caused by it, how to use fossil energy efficiently and cleanly has become a top priority. Fuel cell is the fourth generation of power-generation technology after hydropower, thermal power, and nuclear power, and is a device that directly converts the chemical energy of fuel into electricity. Fuel cells have attracted much attention because of their huge development potential in stationary power stations, portable mobile power sources, and especially in the fields of sustainable energy development. Compared with traditional power-generation devices, fuel cells have high energy conversion efficiency and low emissions [1].

Solid oxide fuel cell (SOFC) is an all-solid-state energy-conversion device that can convert the chemical energy of fuel directly into electrical energy with outstanding advantages such as high efficiency and being environmentally friendly [2]. The operating temperature of conventional SOFC is generally 800–1000 °C, and operating at such high temperatures will bring many problems. Firstly, the choice of materials is limited, such as connector materials made of metal alloys. Secondly, compatibility issues, as reactions can occur between SOFC components at high temperatures. Thirdly, the preparation cost is high [3]. Therefore, the development of intermediate-temperature solid oxide fuel cells (IT-SOFCs) has become a hot topic in current research.

A key obstacle to decreasing the operating temperature of SOFC is the poor catalytic activity towards oxygen reduction reaction (ORR) for the conventional cathodes [4]. Therefore, various new types of IT-SOFC cathodes have been researched extensively, especially mixed ionic and electronic conducting (MIEC) perovskites [5,6,7]. (La,Sr)MnO_3_ (LSM), as a traditional cathode material, has great electrical conductivity and high stability. However, LSM exhibits low catalytic activity and high polarization resistance at intermediate-temperature conditions, making LSM unsuitable as an IT-SOFC cathode material [8,9]. Moreover, LSM is a pure electronic conductor at intermediate-temperature conditions. Thus, the active sites for ORR are restricted in the vicinity of the electrode/electrolyte interfaces [10]. However, the active sites for ORR expand from the electrode/electrolyte interfaces to the electrode body when MIEC perovskites are used as cathode materials, thereby promoting electrochemical performance [11,12].

Recently, cobalt-based perovskite oxides LnBaCo_2_O_5+*δ*_ (Ln = La, Pr, Nd, Sm, Gd, and Y) have been extensively studied as potential cathode materials [13,14,15,16,17,18]. LnBaCo_2_O_5+*δ*_ oxides possess fast oxygen-diffusion and oxygen-surface-exchange properties at intermediate-temperature conditions, as well as good electronic and ionic conductivity, and high oxygen ion permeability, which can effectively extend the region of the three-phase boundary (TPB), thus exhibiting excellent electrochemical activity [19,20]. However, this material also owns some disadvantages. Firstly, a high thermal expansion coefficient (TEC) leads to cracking between SOFC components. Secondly, high Co content makes high cost. It was found that the partial replacement of Co with some transition metals at B sites can effectively solve the above problems [21]. The TEC of SmBaCo_2−*x*_Ni*_x_*O_5+*δ*_ reduced from 19.96 × 10^−6^ K^−1^ to 15.59 × 10^−6^ K^−1^ when the doping amount of Ni was at *x* = 0.3 [22]. The corresponding polarization resistance exhibited a minimum value of 0.0464 Ω cm^2^ at 800 °C. Kim et al. [23] found that the TEC of NdBaCo_2−*x*_Fe*_x_*O_5+*δ*_ reduced from 21.5 × 10^−6^ K^−1^ to 18.3 × 10^−6^ K^−1^ when the doping amount of Fe was at *x* = 2 and the TEC of GdBaCo_2−*x*_Fe*_x_*O_5+*δ*_ reduced from 19.9 × 10^−6^ K^−1^ to 18.8 × 10^−6^ K^−1^ when the doping amount of Fe was at *x* = 1. More recently, it has been reported that the introduction of high-valent cation Mo into the B sites of perovskite oxides can reduce TEC while obtaining good electrochemical performance [24,25]. In the present work, Mo-doped LaBaCo_2_O_5+*δ*_ perovskites were synthesized and evaluated as IT-SOFC cathodes. The effect of Mo content on thermal expansion as well as electrical conductivity and electrochemical performance were investigated.

## 2. Materials and Methods

A sol-gel method was used to prepare LaBaCo_2−*x*_Mo*_x_*O_5+*δ*_ (LBCM*x*, *x* = 0, 0.02, 0.04, 0.06 and 0.08). Raw materials of La(NO_3_)_3_·6H_2_O (99.9%, Sinopharm, Beijing, China), Ba(NO_3_)_2_ (99.5%, Sinopharm, China), Co(NO_3_)_2_·6H_2_O (98.5%, Sinopharm, Beijing, China), and (NH_4_)_6_Mo_7_O_24_·4H_2_O (99.0%, Sinopharm, Beijing, China) were weighed according to stoichiometric amounts and dissolved in distilled water. Then, citric acid (C_6_H_8_O_7_·H_2_O, 99.5%, Sinopharm, China) was added to the solutions. The molar amount of citric acid is 1.5 times that of all metal ions. Aqueous ammonia (NH_3_·H_2_O, 25%, Tongcheng, China) was used to adjust the pH of the solutions to 7. The solutions were heated at 80 °C to form gels, followed by sintering at 500 °C to remove the organics and water to form precursor powders. The precursor powders were finally calcined at 950 °C for 2 h in a furnace to obtain the LBCM powders. The crystal structures of the synthesized powders were characterized by X-ray diffraction (XRD, X’Pert^3^Powder, PANalytical, Almelo, The Netherlands) with Cu Kα radiation. The synthesized powders were pressed into bars and then sintered at 950 °C for 2 h to test the electrical conductivity and thermal expansion coefficient of the LBCM cathodes. The electrical conductivity of the cathode bars was measured via a DC four-probe method in air from 300 °C to 800 °C. The thermal expansion of cathode bars was analyzed by a dilatometer (ZRPY-RY-1400, Xiangtan, China) in air from 25–800 °C.

Commercial 8 mol% yttria-stabilized zirconia (YSZ) powder (Tosoh, Japan) was used to prepare the electrolyte. The YSZ powders were pressed and then sintered at 1500 °C for 5 h in air to yield the dense disks 15 mm in diameter and 0.3 mm in thickness. Symmetrical cells with a configuration of LBCM|YSZ|LBCM were fabricated by a screen-printing method. Cathode slurries containing the LBCM powders and ethyl cellulose and terpineol were screen-printed onto both sides of the YSZ disks and then were sintered at 950 °C for 2 h in air. Ag pastes were used as the current collectors and were painted onto the surfaces of the LBCM cathodes. The electrochemical impedance spectra (EIS) of the symmetrical cells were evaluated from 100 kHz to 0.1 Hz with a signal amplitude of 10 mV under open-circuit conditions via an electrochemical workstation (CHI604E, Chenhua, Shanghai, China). The microstructures of the cross-section were identified by a field emission scanning electron microscope (SEM, Nova NanoSEM 450, FEI, Hillsboro, OR, USA) and an energy dispersive X-ray spectroscopy (EDS, AZtec X-Max^N^80, Oxford Instruments, Oxford, UK).

The performance of single cells with a configuration of LBCM|YSZ|NiO–YSZ was investigated. NiO–YSZ (50 wt.% NiO) anodes were painted onto one side of the YSZ disks, followed by sintering at 1350 °C for 2 h in air. Then, LBCM cathodes were painted onto the other side of the YSZ disks, followed by sintering at 950 °C for 2 h in air. The tests of the single cells were carried out using stationary air as oxidant and hydrogen with a flow rate of 100 mL min^−1^ as fuel. The current-voltage curves were investigated via a DC electronic load (IT8511+, ITECH, Nanjing, China).

## 3. Results and Discussion

### 3.1. Crystal Structure

**Figure 1:**
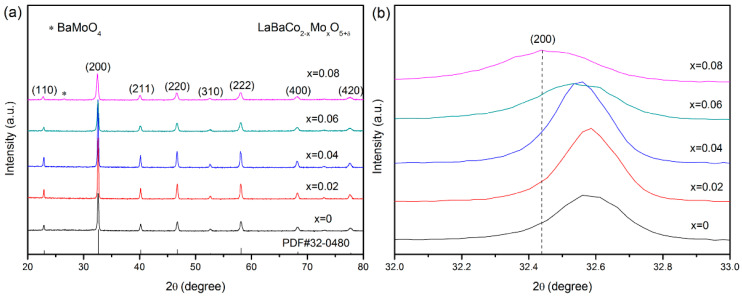
(**a**) XRD patterns of the synthesized LBCM*x* (*x* = 0–0.08) powders. (**b**) Magnification of the patterns in the 2θ range of 32° to 33°.

The XRD patterns of the synthesized LBCM*x* (*x* = 0–0.08) powders are shown in Figure 1a. The crystal structure of the undoped LBC displays a cubic perovskite structure with a *Pm-3m* space group, which is identical to the structure described in the PDF card (#32–0480) and reported by Gu et al. [26]. The crystal structures of LBCM*x* do not change with the incorporation of Mo. However, the second phase BaMoO_4_ appears when the doping amount of Mo reaches 0.08, indicating that the doping amount of Mo in LBC cannot exceed 0.06. A similar result was reported by Xu et al. [24]. They found that the impurity phase BaMoO_4_ was observed in the XRD pattern of PrBaCo_2__−_*_x_*Mo*_x_*O_5+*δ*_ powder when the doping amount of Mo reached 0.07.

The lattice parameters calculated from the patterns are listed in Table 1. The lattice parameters and unit cell volumes (*V*) gradually increase with increasing Mo content, indicating that a lattice expansion appears with the incorporation of Mo, likely due to the substitution of Mo^6+^ with a larger radius (0.59 Å) for Co^3+^ with a smaller radius (0.55 Å). Figure 1b shows the magnification of the patterns in the 2θ range of 32° to 33°. The peak of the (200) plane shifts to a lower angle, which is consistent with the lattice expansion.

### 3.2. Microstructure

**Figure 2:**
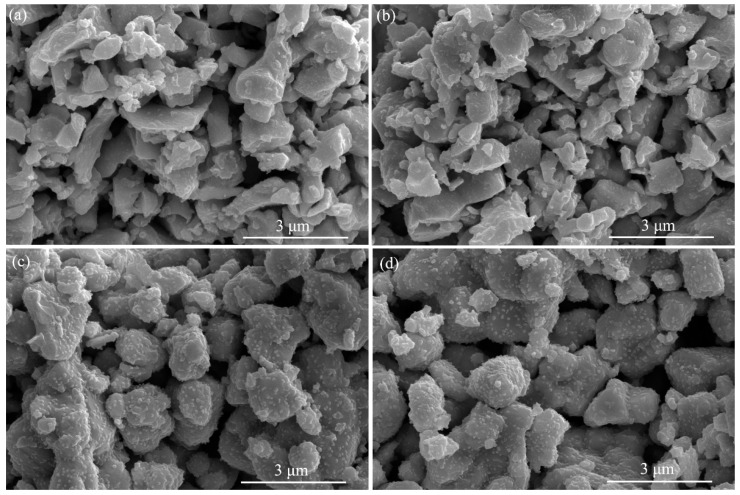
SEM images of LBCM*x* (*x* = 0–0.06) sintered at 950 °C for 2 h: (**a**) *x* = 0, (**b**) *x* = 0.02, (**c**) *x* = 0.04, and (**d**) *x* = 0.06.

**Figure 3:**
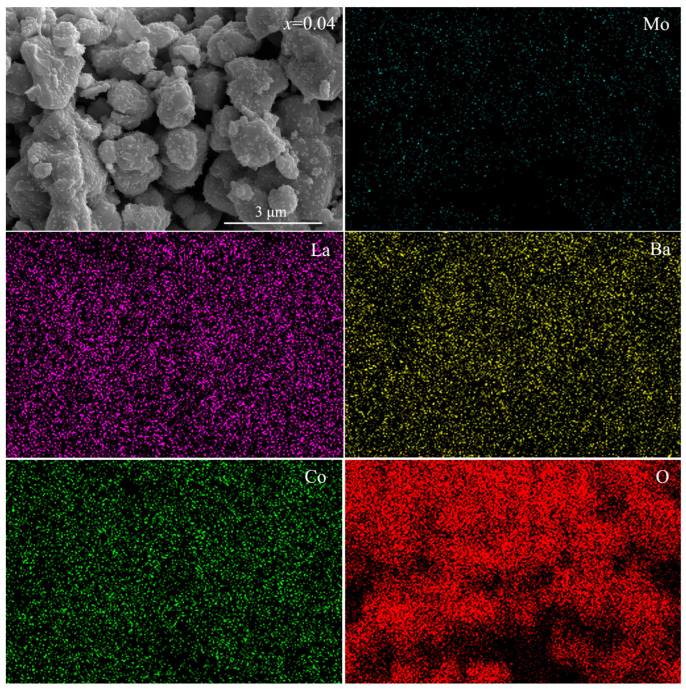
Elements mapping of LBCM0.04.

The SEM images of LBCM*x* (*x* = 0–0.06) sintered at 950 °C for 2 h are shown in Figure 2. All samples exhibit a porous structure with uniform distribution of pores and crystallites. The crystallite size appears to increase slightly with Mo incorporation. When the doping amount of Mo reaches 0.04, nanocrystallites appear on the surface of the cathode crystallites, probably due to cations leaching on the surface of the perovskites after heat treatment. Previous studies reported that the segregation of Ba and Co cations was observed in the B-site doped PrBaCo_2_O_5+*δ*_ systems [27,28]. The elements mapping of LBCM0.04 obtained by EDS is shown in Figure 3. The Mo element is uniformly distributed in the matrix without segregation.

### 3.3. Thermal Expansion

**Figure 4:**
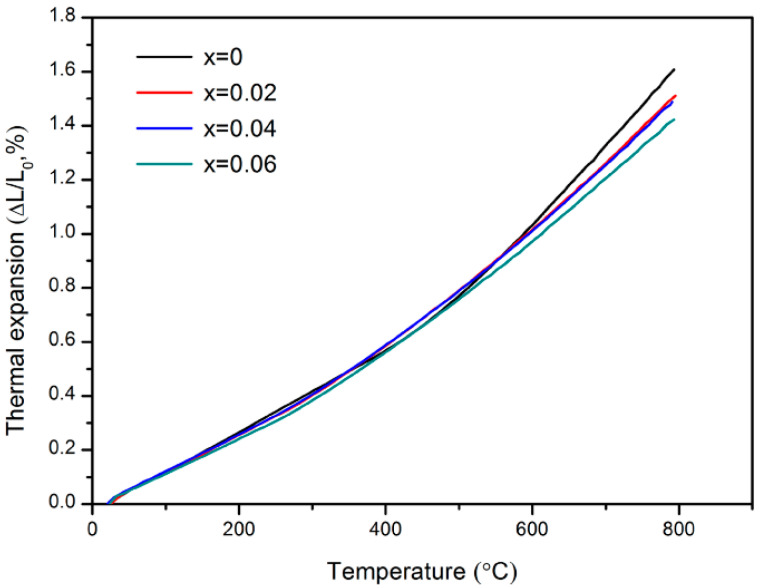
Thermal expansion curves of LBCM*x* (*x* = 0–0.06) from 25 °C to 800 °C.

The thermal expansion curves of the LBCM*x* (*x* = 0–0.06) cathodes from 25 °C to 800 °C are shown in Figure 4. The thermal expansion of LBCM*x* displays a linear increase with the elevated temperature. For cobalt-based calcite materials, the spin state of Co cation is affected by temperatures. As the temperature increases, Co^3+^ transitions from a low-spin state ($t_{2g}^{6}e_{g}^{0}$) to a high-spin state ($t_{2g}^{4}e_{g}^{2}$), and the corresponding ionic radius also transitions from 0.55 Å in the low-spin state to 0.61 Å in the high-spin state, thereby increasing the thermal expansion [29].

The thermal expansion coefficients (TECs) calculated from the curves are listed in Table 2. Doping Mo reduces the TECs of the samples from 20.87 × 10^−6^ K^−1^ to 18.47 × 10^−6^ K^−1^. It can be known from the Grüneisen law:

(1) $$\alpha_{v}=\frac{\lambda C_{v}}{VB}$$

where *α_v_* is the volume expansion coefficient, *λ* is the Grüneisen constant, *C_v_* is the heat capacity of the volume constant, *V* is the unit cell volume, and *B* is the bulk elastic modulus that the volume expansion coefficient decreases with increasing the unit cell volume [30]. According to the XRD results, doping Mo increases the unit cell volume of the samples. Moreover, the substitution of Mo for Co and the consequent reduction in the Co content in LBCM leads to the reduction of oxygen vacancies, thereby resulting in a decrease in TECs [23]. Similar thermal expansion behaviors were reported in B-site Mo-doped La_0.6_Sr_0.4_Co_0.2_Fe_0.8_O_3−*δ*_ systems [31].

### 3.4. Electrical Conductivity

**Figure 5:**
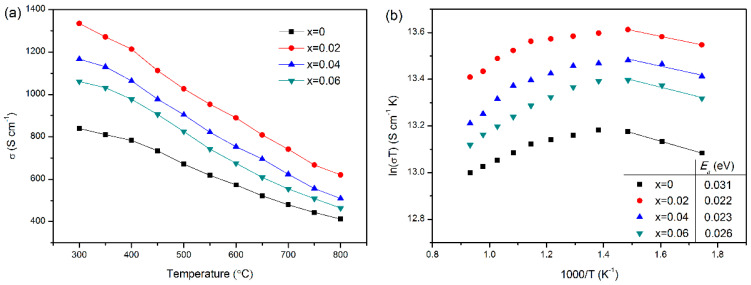
(**a**) Electrical conductivity and (**b**) Arrhenius plots of LBCM*x* (*x* = 0–0.06) as a function of temperature.

Figure 5a shows the electrical conductivity of LBCM*x* (*x* = 0–0.06) as a function of temperature. The conductivity of all samples decreases with increasing temperature, which is consistent with a small-polaron hopping mechanism. Similar results were found by PrBa_0.5−*x*_Sr_0.5_Co_2_O_5+*δ*_ [32] and NdSrCo_2_O_5+*δ*_ [33] perovskites. The incorporation of Mo improves the conductivity of LBC, which may be attributed to the decrease in the concentration of oxygen vacancies accompanied by doping Mo, thereby reducing the barriers to electron transport. However, the electrical conductivity decreases when the Mo content exceeds 0.02, which may be related to the decrease in the concentration of small polaron by the incorporation of poorly conductive Mo cation. The conductivity of all samples is above 400 S cm^−1^ in the operating temperature range of 600–800 °C, which meets the requirements of IT-SOFC for the conductivity of cathode materials (>100 S cm^−1^).

Figure 5b shows the Arrhenius plots of the electrical conductivity for LBCM*x* (*x* = 0–0.06) from 300–800 °C. There is a linear relationship between ln(*σT*) and 1000/*T* of all samples at low temperatures, suggesting that the conductive behavior of LBCM*x* is consistent with a small-polaron hopping mechanism and satisfies the following equation:

(2) $$\sigma =\frac{A}{T}\exp\left(\frac{-E_{a}}{kT}\right)$$

where *σ* is the conductivity, *A* is the pre-exponential factor, *T* is the absolute temperature, *k* is the Boltzmann constant, and *E_a_* is the conductivity activation energy. The values of *E_a_* obtained by fitting the data points at the low-temperature region are listed in Figure 5b. After doping Mo at the B-site, the *E_a_* of the samples was reduced, indicating that the addition of appropriate Mo cation improves electron transport due to the decrease in the concentration of oxygen vacancies. Wang et al. [34] reported a linear relationship between ln(*σT*) and 1000/*T* for Sr_2_Co_1−*x*_Nb*_x_*FeO_5+*δ*_ cathodes at low temperatures, and the activation energy was reduced from 13.4 kJ mol^−1^ to 13.1 kJ mol^−1^ after doping Ni cation at *x* = 0.1.

### 3.5. Electrochemical Impedance

**Figure 6:**
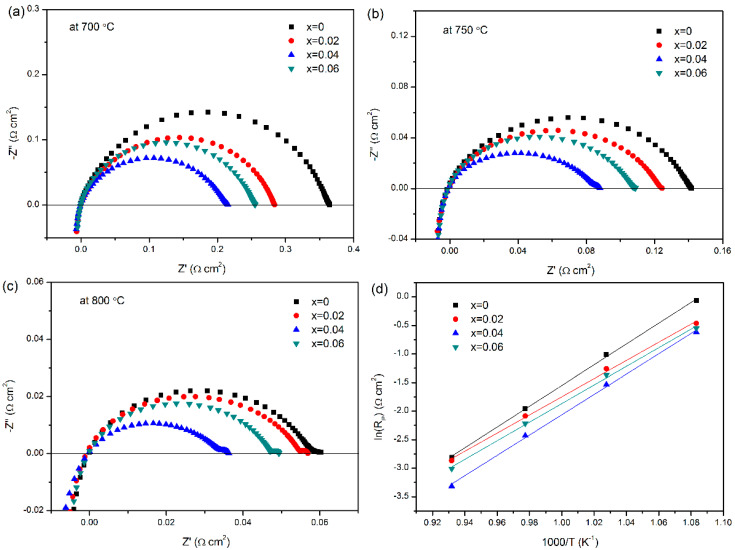
EIS of the LBCM*x* (*x* = 0–0.06) cathodes at (**a**) 700 °C, (**b**) 750 °C, and (**c**) 800 °C under open-circuit conditions. (**d**) Arrhenius plots of the *R*_P_ for LBCM*x* (*x* = 0–0.06) from 600 °C to 800 °C.

The EIS of the LBCM*x* (*x* = 0–0.06) cathodes at 700–800 °C under open-circuit conditions are shown in Figure 6. The EIS were fitted by the equivalent circuit of *LR*_ohm_(*Q*_1_*R*_1_)(*Q*_2_*R*_2_). The meaning of each parameter in the equivalent circuit was described in the previous article [35]. The ohmic resistance (*R*_ohm_) was removed from the EIS for comparison purposes. With the increase in Mo doping amount, the polarization resistance (*R*_P_, the sum of *R*_1_ and *R*_2_) of the cathodes decreases. When the doping amount *x* of Mo increases to 0.04, the *R*_P_ of the cathode reaches the minimum value. The corresponding *R*_P_ at 700–800 °C is 0.216 Ω cm^2^, 0.088 Ω cm^2^, and 0.036 Ω cm^2^, reduced by a factor of 1.69, 1.61, and 1.67, respectively, when compared with the undoped LBC. Previously, it was found that Mo doping could facilitate the decomposition and diffusion of oxygen in the three-phase boundary (TPB), which strongly improved the electrochemical redox reaction of the cathodes [36]. However, the *R*_P_ increases slightly instead when the content of Mo is further increased to 0.06. The reduction reaction of oxygen by the MIEC cathode occurs not only at the TPB but also within the cathode body and on the cathode surface. The adsorbed oxygen atoms formed by the adsorption and dissociation of oxygen molecules at the cathode/air interface are reduced to oxygen ions in the cathode body. The oxygen vacancies in the cathode body can provide channels for the migration of oxygen ions, and the oxygen vacancies on the surface are the active sites for the exchange reactions of surface oxygen species. Thus, high oxygen vacancy concentrations in the cathode can greatly facilitate the diffusion of oxygen ions in the material body and may provide more surface-active sites needed for ORR, thereby promoting the charge transfer in the cathode body and the adsorption/decomposition of oxygen on the cathode surface, and consequently leading to a decrease in the polarization loss. However, in order to maintain the electrical neutrality of the crystal, the substitution of high-valent Mo for the low-valent Co may lead to the reduction of the B-site cation or the decrease of the oxygen vacancy. Based on the analysis of the above TECs results, the decrease in oxygen vacancy concentration may play an important role when a high level of Mo is doped. As a result, the increase in *R*_P_ with a further increase in Mo doping (x = 0.06) may be attributed to the decrease in oxygen vacancy concentration.

Figure 6d shows the Arrhenius plots of the *R*_P_ for LBCM*x* (*x* = 0–0.06) from 600 °C to 800 °C. The activation energy *E_a_* calculated from the slope of the fitting curve for the LBCM*x* cathodes at *x* = 0, 0.02, 0.04, and 0.06 is 1.57 eV, 1.37 eV, 1.53 eV, and 1.40 eV, respectively, which are close to the activation energy of previously reported PrBaCo_2−*x*_Fe*_x_*O_5+*δ*_ [37], NdBa_1−*x*_Sr*_x_*Co_2_O_5+*δ*_ [38], and LnBa_0.5_Sr_0.5_Co_1.5_Fe_0.5_O_5+*δ*_ [39]. It can be seen in the present study that the Mo-doped cathodes exhibit lower activation energy, indicating that LBCM*x* (*x* = 0.02–0.06) possess a better catalytic activity for ORR. The lower activation energy in the Mo-doped cathodes may be due to the suppression of oxygen vacancies by Mo doping at the B-site that restores the hole transport network, enhances mobility, and reduces the activation energy. A similar result was reported by Baijnath et al. where the activation energy of Ca_2_Fe_2_O_5_ was reduced from 2.70 eV to 1.35 eV by doping with Mo [40].

In order to evaluate the mechanism of introducing Mo on the electrochemical performance of the cathode, the *R*_1_ and *R*_2_ values calculated from the fitted EIS are listed in Table 3. For the MIEC cathode materials, high-frequency arc *R*_1_ reflects the charge transfer of ORR and includes the electron and oxygen ion transport processes that occur on the electrode surface and the electrode/electrolyte contact surface. In addition, the process of cathode-adsorbed O_2_ gaining electrons into O^2−^ is also closely related to high-frequency charge transfer. The low-frequency arc *R*_2_ mainly represents the adsorption and dissociation of oxygen on the cathode surface, as well as the bulk diffusion processes. For all cathode materials in this study, the high-frequency arc *R*_1_ is much higher than the low-frequency arc *R*_2_, indicating that the charge transfer process is the main limiting factor for the cathodic ORR. The proportion of *R*_1_ to *R*_P_ decreases after the incorporation of Mo, suggesting that Mo mainly enhances the charge transfer process, which is attributed to the increased conductivity.

### 3.6. Single-Cell Performance

**Figure 7:**
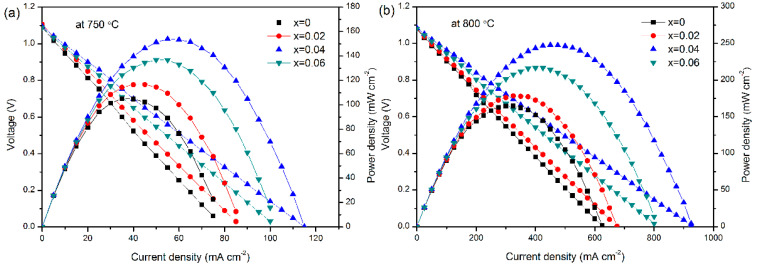
Output performance of the single cells with LBCM*x* (*x* = 0–0.06) cathodes at (**a**) 750 °C and (**b**) 800 °C.

The output performances of the single cells with LBCM*x* (*x* = 0–0.06) cathodes at 750 °C and 800 °C are shown in Figure 7. The maximum power density of the for the LBCM*x* cathodes at *x* = 0, 0.02, 0.04, and 0.06 is 105, 117, 154, and 137 mW cm^−2^ at 750 °C, respectively, and 165, 178, 248, and 217 mW cm^−2^ at 800 °C, respectively. The single-cell performance is significantly improved after doping with Mo. The optimal single-cell performance was achieved when the doping amount of Mo was 0.04. The above results are consistent with those of the EIS, indicating that Mo doping is an effective method to enhance the electrochemical performance of LBC cathode at intermediate-temperature conditions.

## 4. Conclusions

Mo-doped LBC (LBCM*x*) were synthesized and assessed as cathodes for IT-SOFCs. The Mo incorporation did not change the crystal structure of the sample, which still showed a cubic perovskite structure with a *Pm-3m* space group. The solid solubility of Mo in LBCM was lower than 0.08, otherwise the second phase BaMoO_4_ would appear. When the doping amount of Mo increased from 0 to 0.06, the TEC decreased from 20.87 × 10^−6^ K^−1^ to 18.47 × 10^−6^ K^−1^. Mo doping improved the cathodic conductivity, showing a conductivity higher than 400 S cm^−1^ between 600–800 °C. The electrochemical performance of the samples was also enhanced after Mo doping and the optimal electrochemical performance occurred at a doping level of 0.04. The corresponding polarization resistance and maximum power density at 800 °C was 0.036 Ω cm^2^ and 248 mW cm^−2^, respectively.

## Figures and Tables

**Table 1:** Lattice parameters and unit cell volumes of LBCM*x*.

	Space Group	*a* (Å)	*b* (Å)	*c* (Å)	*V* (Å)^3^
*x* = 0	*Pm-3m*	5.4869	5.4869	5.4869	165.1890
*x* = 0.02		5.4890	5.4890	5.4890	165.3787
*x* = 0.04		5.4913	5.4913	5.4913	165.5867
*x* = 0.06		5.4916	5.4916	5.4916	165.6139
*x* = 0.08		5.7123	5.7123	5.7123	186.3945

**Table 2:** TECs of LBCM*x*.

	*x* = 0	*x* = 0.02	*x* = 0.04	*x* = 0.06
TEC (×10^−6^ K^−1^)	20.87	19.61	19.30	18.47

**Table 3:** *R*_1_, *R*_2,_ and *R*_P_ values of LBCM*x* (Units: Ω cm^2^).

	700 °C			750 °C			800 °C		
	*R*_1_	*R*_2_	*R*_P_	*R*_1_	*R*_2_	*R*_P_	*R*_1_	*R*_2_	*R*_P_
*x* = 0	0.338 (93%)	0.027 (7%)	0.365	0.136 (96%)	0.006 (4%)	0.142	0.057 (95%)	0.003 (5%)	0.060
*x* = 0.02	0.258 (91%)	0.026 (9%)	0.284	0.117 (94%)	0.007 (6%)	0.124	0.053 (93%)	0.004 (7%)	0.057
*x* = 0.04	0.194 (90%)	0.022 (10%)	0.216	0.080 (91%)	0.008 (9%)	0.088	0.032 (89%)	0.004 (11%)	0.036
*x* = 0.06	0.237 (93%)	0.019 (7%)	0.256	0.103 (95%)	0.006 (5%)	0.109	0.046 (94%)	0.003 (6%)	0.049

## Data Availability

Not applicable.
